# Broadly neutralizing antibodies for treatment of HIV in children in the Global South: rationale and challenges

**DOI:** 10.3389/fped.2026.1833681

**Published:** 2026-07-23

**Authors:** Alfredo Tagarro, Arantza Cobela-García, Shelly Malhotra, Katerina Chapman, Pablo Rojo

**Affiliations:** 1 Innovation in Global Paediatric Infectious Diseases Research Group, Instituto de Investigación 12 de Octubre (imas12), Madrid, Spain; 2 Department of Pediatrics, Infanta Sofía University Hospital. Fundación Para la Investigación Biomédica e Innovación Hospital Universitario Infanta Sofía y Hospital del Henares (FIIB HUIS HHEN), Madrid, Spain; 3 Department of Medicine, Faculty of Medicine, Health and Sports, Universidad Europea de Madrid, Madrid, Spain; 4 CIBER in Infectious Diseases (CIBERINFEC), Instituto de Salud Carlos III, Madrid, Spain; 5 IAVI, New York, NY, United States; 6 Universidad Complutense de Madrid, Madrid, Spain

**Keywords:** bNAB, children, clinical trials, HIV, treatment

## Abstract

Despite the rapid scale-up of early infant diagnosis and antiretroviral therapy, pediatric HIV type 1 (HIV-1) remains a remarkable public health as children account for 15% of all AIDS-related deaths worldwide. Broadly neutralizing antibodies (bNAbs) have emerged as a promising strategy for the prevention, treatment, and potential cure of HIV. In adults, bNAbs have proven to be safe and effective, capable of suppressing plasma viremia and delaying viral rebound during analytical treatment interruptions. bNAbs provide immediate passive immunity and long-acting coverage, which makes them suitable for children where high baseline viral loads drive rapid disease progression and reservoir establishment. Several trials have demonstrated that subcutaneous administration of bNAbs is safe and pharmacokinetically feasible in neonates and have provided a proof-of-concept for bNAb-mediated viral control in children. However, the clinical evaluation of bNAbs has primarily been directed towards adult populations in the Global North, leading to limited pediatric clinical data. This research lag represents a critical ethical inequity that risks denying life-saving therapeutics to the most vulnerable populations. Therefore, equitable access in the Global South requires the elimination of structural barriers that currently delay pediatric access through a strategy of intentional early inclusion, technology transfer, and parallel clinical development. Similarly, a multi-faceted approach involving governments in the Global South, funders, stakeholders, and pharmaceutical developers is essential to address intellectual property sharing, licensing, regulatory harmonization, and local manufacturing. The ongoing development of pediatric trials represents an opportunity to achieve long-term viral remission, providing a definitive pathway to end the pediatric HIV epidemic. Prioritizing these studies is a scientific and moral necessity to ensure that children are finally placed at the center of medical innovations and no longer excluded from therapeutic advancements.

## 1 Introduction

The pediatric Human Immunodeficiency Virus (HIV) pandemic remains a significant public health challenge in the Global South. Of the estimated 1.4 million children living with HIV (CLHIV), 83% of the total cases are concentrated in Sub-Saharan Africa (1). The current standard of care presents several obstacles in resource-limited settings that exacerbate this epidemiological crisis, including the lack of palatable pediatric formulations, the necessity of life-long adherence, the associated medication fatigue, and the social stigma associated with daily pill-taking. To address these structural and clinical barriers, novel long-acting strategies are required. Unlike traditional antiretrovirals (ARVs), which may require months or years to achieve full suppression in children (2), broadly neutralizing antibodies (bNAbs) provide immediate passive immunity. By decreasing HIV viral load (VL) during the critical first months of life, bNAbs can help reduce the inflammatory milieu and limit the morbidity and mortality associated with high viremia.

bNAbs are potent monoclonal antibodies characterized by their ability to neutralize multiple HIV type 1 (HIV-1) isolates at low concentrations (3). bNAbs target several conserved epitopes on the HIV-1 envelope trimer, including the CD4 binding site, V3-glycan, V1/V2-glycan, Membrane-Proximal External Region (MPER), fusion peptide, gp120-gp41 interface, and silent-face (4). bNAbs are isolated from a minority of HIV-1 elite neutralizers who naturally develop antibodies exhibiting exceptional breadth and potency against multiclade HIV-1 subtypes (5–8). These antibodies are often ineffective in the individual who produces them due to viral escape through mutations. However, when isolated and provided to other people, they can be a promising option for prevention, treatment, and potential cure of HIV infection.

Beyond direct neutralization, bNAbs prevent viral dissemination by enhancing antibody-dependent cellular cytotoxicity (ADCC), opsonization and innate immune activation (9, 10). Due to this combined antiviral effect bNAbs can significantly reduce the VL, and the intact proviral reservoir. Bioengineering has further improved the efficacy and stability of these bNAbs. For instance, VRC07 has a heavy-chain sequence that shares 90% nucleotide sequence identity with VRC01 but differs at 15 amino acid positions and contains a four-amino-acid insertion in the heavy-chain third complementarity determining region (CDR H3) (11, 12). This difference led to more potency than VRC01 (11, 12). Importantly, the introduction of the -LS mutation (M428L/N434S) results in an increase in the half-life of bNAbs by enhancing binding to the neonatal receptor and preventing acidic degradation (13), opening the possibility of administration every three to six months. For instance, the estimated half-life of PGT121.414.LS was three times that of its parental form, PGT121 (14). Consequently, bNAbs have emerged as a transformative alternative that could facilitate long-term viral remission.

## 2 Clinical trials in adults

The initial fist in human (FIH) study of bNAbs was a Phase I dose-escalation trial to evaluate VRC01 safety in healthy humans, published in 2015 by Ledgerwood et al. (16). The first demonstration of antiviral efficacy in people living with HIV (PLHIV) came from the studies published by Lynch et al. (17), Caskey et al. (18), Bar et al. (19), and Scheid et al. (20) (Table 1).

**Table 1:** Summary of clinical trials evaluating the virological efficacy of bNAb monotherapy, combination regimens, and bNAb-ARV strategies in adults. SC, subcutaneous; IV, intravenous.

bNAbs	NCT	Phase	doses	Efficacy	Reference
Alone:					
3BNC117	NCT02018510	I	1	VL reduction in 0.8–2.5 log	Caskey et al., 2015. Nature (18)
VRC01	NCT01950325	I	1	VL reduction in 1.1–1.8 log	Lynch et al.*, 2015.* Sci Transl Med (17)
VRC01	NCT02471326	I	1	Viral suppression up to 8 weeks following ATI	Bar et al., 2016. N Engl J Med (19)
3BNC117	NCT02446847	II	2, 4	Viral suppression up to 7–10 weeks following ATI	Scheid et al., 2016. Nature (20)
10-1074	NCT02511990	I	1	VL reduction in 1.52 log	Caskey et al., 2017. Nat Med (21)
VRC01	NCT02664415	II	1	Time to rebound after ATI 33 days	Crowell et al., 2019. Lancet (32)
PGT121	NCT02960581	I	1	VL reduction in 1.77 log	Stephenson et al., 2021. Nature (22)
N6LS	NCT04871113	II	1	VL reduction in 0.43–1.72 log	Leone et al., 2025. J Infect Dis (24)
VRC01-LS or VRC07-523LS	NCT02840474	I	1	VL reduction in 0.5–2.6 log	Happe et al., 2025. JCI Insight (25)
In combination:					
3BNC117 + 10-1074	NCT02825797	I	1, 3	Viral suppression up to 21 weeks following ATI	Mendoza et al., 2018. Nature (29)
3BNC117 + 10-1074	NCT03571204	I	8	Maintain suppression up to 32–29 weeks following ATI	Gaebler et al., 2022. Nature (33) Sneller et al., 2022. Nature (34)
VRC07-523LS + PGT121 + PGDM1400	NCT03721510	I/II	1, 3	VL reduction in 1.5–2.21 log	Julg et al., 2022. Nat Med (28)
3BNC117 + 10-1074	NCT04319367	II	2	Maintain suppression up to 20–72 weeks following ATI	Fidler et al., CROI #107 2025 (35)
With ARVs:					
3BNC117 + 10-1074LS	NCT05729568	I	1	Maintain suppression up to 26 weeks	Eron et al., CROI #193 2023 (27)
N6LS + Cabotegravir	NCT05996471	II	6	Maintain suppression up to 6 months	Leone et al., CROI #203 2025 (26)

These trials demonstrated that the administration of bNAbs in PLHIV is safe, and effective in maintaining viral suppression and delaying viral rebound during antiretroviral treatment interruptions (ATI). Early phase I trials demonstrated that infusion of 3BNC117 (18, 20), 10-1074 (21), PGT12 (22), or N6LS (23, 24) could transiently reduce viremia by 0.5 to 2.5 log copies/mL. The field further evolved with the incorporation of the -LS mutation, allowing for less frequent dosing as bNAbs tissue retention and persistence improved (25). Recent studies have successfully administered these long-acting antibodies with ARVs (26, 27), such as Cabotegravir, or combination of bNAbs (14, 28–31) to achieve sustained viral control.

bNAbs have been tested not only for treatment, but also for prevention and cure. Regarding prevention, the AMP trials (HVTN 704/HPTN 085) showed the potential of VRC01 for HIV-1 prevention in adults but raised the issue of susceptibility (15). Although the AMP-study provided proof-of-concept that VRC01 could reduce the acquisition of sensitive HIV-1 isolates, its overall efficacy was limited by pre-existing viral resistance. Pre-trial modeling suggested that 65%–81% of regional isolates would be sensitive to VRC01, but only 30% of circulating strains were effectively inhibited by VRC01. Despite these limitations, VRC01 showed a 75.4% efficacy at preventing HIV-1 infection, but only for isolates that were sensitive (IC_80_ < 1 μg/mL). Moreover, the AMP trial identified serum neutralization titers as the first correlate of protection for antibody-based interventions (36).

Regarding cure, the RIO trial also provided a significant milestone in bNAb research, demonstrating that dual therapy with long-acting antibodies could serve as a cornerstone for HIV-1 remission strategies. In this study, the administration of 3BNC117-LS and 10-1074-LS effectively prevented viral rebound during analytical treatment interruption (ATI) (35). Viral suppression was maintained in 75% of the participants 20 weeks after ART interruption and in 39% of the participants for more than 72 weeks after ATI. These findings provided a proof-of-concept for the role of bNAbs in achieving long-term viral remission and HIV-1 cure (35).

These trials reinforce the potential of bNAbs as a viable intervention for achieving prevention, viral suppression, and cure in adults living with HIV. However, due to the distinct transmission dynamics, distribution volume, metabolism, growth, and other factors observed in children, the direct extrapolation of data to pediatric cohorts from preclinical trials and trials in adults remains limited. Although the use of non-human primate (NHP) models has been essential to provide the first evidence that passive immunization of bNAbs could prevent simian-human immunodeficiency virus (SHIV) transmission (37), HIV-1 is inherently more aggressive and genetically diverse than the SHIV strains used in NHPs (38). Unlike adults, pediatric HIV-1 infection may involve cell-associated transmission, which requires significantly higher antibody concentrations for effective neutralization than cell-free virus (39). These biological and physiological disparities, along with regulatory requirements, highlight the critical need for pediatric trials.

## 3 Rationale for treatment with bNAbs in children

Mortality rates among CLHIV are alarmingly high, reflecting the aggressive clinical course of the virus in this population. Without intervention, approximately 50% of CLHIV die before their second birthday, with mortality reaching 80% by the age of five (2). This burden persists despite early ART administration in infants diagnosed at birth where mortality within the first year of life remains over 10% and can increase to 45% in those with advanced HIV disease.

The strongest risk factor for these adverse outcomes is the infant baseline VL (2). The first months of life represent a critical window where viremia drives rapid disease progression and early establishment of a persistent viral reservoir. Consequently, interventions that can rapidly decrease VL during this narrow window of opportunity are essential to improve survival. While prevention of Mother-to-Child Transmission (PMTCT) initiatives and ART availability have significantly reduced pediatric incidence reducing intrauterine and intrapartum infection (40), vertical transmission remains the primary mode of infection, particularly during the high-risk breastfeeding and perinatal period (41, 42). In settings where maternal viral suppression is not achieved or sustained at delivery, the current standard of care is often inadequate to protect the infant. In these scenarios, intensification, and feasible interventions such as bNAbs are essential to diminish morbidity and mortality by offering immediate passive immunity.

bNAbs offer several advantages in the pediatric context. As long-acting injectables with favorable safety profiles across all age groups, they are well-suited for administration during the first year of life, requiring only small doses. This is especially relevant for populations facing adherence challenges to oral ART and offers a short-term intervention with potential lifelong benefits. Furthermore, caregiver receptivity to bNAb-based regimens appears remarkably high, often exceeding the acceptance of conventional daily oral ART (43). From a public health perspective, bNAbs are also projected to be cost-effective (44), and have contributed to the prevention of vertical transmission when administered to HIV-exposed infants. Moreover, the transition to subcutaneous administration, recently validated in phase I/II trials for VRC01, VRC01-LS, and N6LS, represents a critical prerequisite for the large-scale implementation of these biologics in resource-limited settings. This makes CLHIV an ideal population for exploring not only bNAb-mediated viral control but also the potential for a functional cure (45).

## 4 Clinical trials in children

Infants living with HIV (ILHIV) who initiate ART from birth represent an optimal population for bNAb-mediated interventions. Early ART can limit the size of the intact proviral reservoir and restrict the emergence of escape mutations, which may enhance the capacity of bNAbs to maintain long-term viremia control (46). Furthermore, the pediatric immune system, characterized by the dominance of the innate immunity during the first years of life, might offer a unique ontogeny that may be more receptive to immunomodulatory therapies. This is supported by the important role that natural killer (NK) cells have in early viral control in infants (47). By exerting cytotoxic functions comparable to those of CD8^+^ T-cells, NK cells might contribute to bNAb-mediated ADCC. Unlike adults, infants present a lower likelihood of pre-existing resistance to bNAbs, supporting their use as an intensification strategy following vertical transmission (48).

Despite their potential, the clinical application of bNAbs in children remains at an early stage, with most evidence derived from phase I or II trials (Table 2).

**Table 2:** Current landscape of pediatric clinical trials investigating bNAbs for HIV-1 prevention, treatment intensification, and long-term viral remission.

Purpose	Study name	Agent	Administration route	Target population	Sample size	Phase	Primary Endpoint	Safety Profile	Local AEs (*n*, %)	Systemic AEs/Laboratory Abnormalities (*n*, %)	Reference
Prevention	IMPAACT P1112 (NCT02256631)	VRC01	Single dose of 20, or 40 mg/kg SC, or 40 mg/kg SC followed by monthly 20 mg/kg SC for 6–18 months	HIV-exposed neonates < 5 days old	*n* = 39	I - Completed	Safety, PK	Well tolerated, Mild to moderate local injection site reaction.	Grade 1/2 = 27, 69% (induration, erythema)	Grade 1 = 4, 10% (fever, decreased appetite and sleep, irritability)	Cunningham et al., 2020. J Infect Dis. (51)
		VRC01LS	Single dose of 80 mg SC, or 80 mg SC followed by 100 mg SC after 12 weeks (total of 2 doses)		*n* = 20				Grade 1 = 15, 75% (erythema)	Grade 1 = 4, 20% (fever, increased sleep, decreased appetite, irritability)	McFarland et al., 2021. J Infect Dis. (50)
		VRC07-523LS	Single dose of 80 mg SC, or 80 mg SC followed by 100 mg/kg SC after 12 weeks (total of 2 doses)		*n* = 20				Grade 1 = 10, 45% Grade 2 = 3, 14% (induration, erythema, edema, tenderness)^d^	Grade 1 = 4, 18% (increased sleep, decreased appetite and sleep, irritability, vomiting)	Cunningham et al., 2025. J Pediatric Infect Dis Soc. (49)
	PedMab1 (PACTR202205715278722)	CAP256V2LS	5, 10, or 20 mg/kg SC	HIV-exposed neonates	*n* = 24	I - Completed	Safety, PK	Well tolerated, Mild to moderate AEs.	Grade 1 = 1, 4% (erythema, induration)	Grade 1 = 2, 8% (elevated AST, fussiness)	Goga et al., 2024. BMC Infect Dis. (66)
		VRC07-523LS	20, or 30 mg/kg SC		*n* = 16				Grade 1 = 7, 44% (erythema, induration)	Grade 1/2 = 1, 6% (elevated AST, fussiness)	
		CAP256V2LS + VRC07-523LS	60 mg/kg SC CAP256V2LS SC and 90 mg SC VRC07-523LS, or 120 mg SC CAP256V2LS, and 120 mg VRC07-523LS		*n* = 8				Grade 1 = 4, 25% (bilateral pain)	Grade 2 = 3, 19% (irritability, anemia, neutropenia) Grade 3/4 = 1, 6% (neutropenia)	
	IMPAACT P2037 (NCT06517693)	PGT121.414.LS	Single dose of 80^c^ mg SC, or 80^c^ mg SC followed by 100 mg SC after 12 weeks (total of 2 doses)	HIV-exposed infants	*n* = 24	I - Recruiting	Safety, PK	Pending	^b^	^b^	-
		PGT121.414.LS + VRC07-523LS	Single dose of 80^c^ mg SC, or 80^c^ mg SC followed by 100 mg SC after 12 weeks (total of 2 doses)		*n* = 24						
Treatment	Tatelo (NCT03707977)	VRC01-LS	30 mg/Kg IV followed by 15 mg/Kg IV every 4 weeks (total of 3 doses)	CLHIV on ART 24 weeks to 12 years old	*n* = 6	I/II -Completed	Safety, PK, Viral Suppression	Well tolerated, No infusion reactions, No grade > 4 AEs.	Grade 1 = ^a^(eczema) Grade 2 = ^a^(dermatitis)	Grade 1 = ^a^(coughing)	Capparelli et al., 2022. J Acquir Immune Defic Syndr. (54)
		10-1074	30 mg/Kg IV every 4 weeks (total of 3 doses)		*n* = 6				None	Grade 1 = 1, 17% (elevated creatinine)	
		VRC01LS + 10-1074	Step1: 30 mg/kg IV followed by 15 mg/kg IV for VRC01LS and 30 mg/kg IV for 10-1074 every 4 weeks (total of 8 doses)	CLHIV on ART	*n* = 28				None	Grade 1/2 = 4, 67% (hypoglycemia, neutropenia)	
			Step 2: 15 mg/kg IV for VRC01LS and 30 mg/kg IV for 10-1074 every 4 weeks (total of 6 doses)	CLHIV off ART	*n* = 25^b^				None	Grade 3 = 3, 12% (neutropenia)	Ajibola et al., 2021. Sci Transl Med. (67)
Cure/Remission	IMPAACT P1115 (NCT02140255)	VRC01	40 mg/kg SC every 4 weeks (total of 4 doses)	CLHIV < 48 h of age	*n* = 11	I/II - Recruiting	HIV Remission	Well tolerated, Rare local injection reactions.	None	None	McFarland *et al*., 2026. CROI P840. (65)
		VRC07-523LS	40 mg/kg SC every 4 weeks (total of 5 doses)		*n* = TBD			Pending	^b^	^b^	-
	IMPAACT 2008 (NCT03208231)	VRC01	40 mg/kg SC every 2–4 weeks (total of 4 doses)	CLHIV on ART ≤ 12 weeks old	*n* = 30	I/II - Completed	Safety, Antiviral Activity	Well tolerated, Mild to moderate local injection site reaction.	Grade 1/2 = 27, 90% (edema, induration)	^b^	Khaitan et al., 2022. AIDS Oral Presentation. (55)
	Tatelo Plus (IMPAACT 2042) (NCT06508749)	PGT121.414-LS + VRC07-523LS + PGDMI400LS	20 mg/kg IV	CLHIV on ART 24 weeks to 12 years old	*n* = 12	I/II - Recruiting	Safety, Viral suppression	Well tolerated, No infusion reactions, No grade ≥ 3 AEs.	None	None	Capparelli et al., (64). CROI P843.
			25 mg/kg IV VRC07-523LS, 25 mg/kg IV PGDMI400LS, and 20 mg/kg IV PGT121.414-LS	CLHIV off ART	*n* ≤ 41			Pending	^b^	^b^	-

a AE not reported per participant.

b Data not available at the time of publication.

c 80 mg ≤ 3 kg, 100 mg > 3 kg at birth.

d Participants recruited from Step 1.

The earliest evidence for the prophylactic potential of bNAbs in children originated from IMPAACT P1112 (49–51), a dose-escalating phase I study that served as the first-in-infant trial to evaluate subcutaneous administration of VRC07-523LS (49), VRC01LS (50), and VRC01 (51) in HIV-exposed newborns. PK data from these trials confirmed that subcutaneous weight-banded dosing could successfully achieve and maintain protective bNAb serum thresholds for several weeks. Similarly, the PedMab study investigated the preventive role of subcutaneous administration of CAP256V2LS and VRC07-523LS in HIV-exposed infants (52). These trials validated the feasibility of passive immunization in neonates and provided the essential pharmacological blueprint for all subsequent pediatric bNAb investigations. However, to date, there are no trials evaluating prevention of vertical transmission through passive immunization of the mother.

The most robust clinical evidence to date for bNAb-mediated viral control comes from the Tatelo study (45, 53, 54), a phase I/II trial that evaluated intravenous administration of VRC01LS and 10-1074 alone or in combination in early-treated, suppressed CLHIV. Following an eight-week overlap with standard ART, participants entered a bNAb-only maintenance phase where ART was discontinued for up to 24 weeks (53). Viral suppression was achieved in 44% of children receiving dual therapy during ATI and remained below 40 copies/mL through the whole phase. Genotypic analysis of provirus in the successful cohort indicated that dual bNAb susceptibility, particularly to 10-1074 was strongly associated with maintained control. Furthermore, the absence of anti-drug antibodies (ADAs) reinforced the favorable immunogenicity profile of these biologics in pediatric populations. While 14 participants experienced viral rebound during the ATI, re-suppression was achieved upon re-initiation of ART. These findings validated the safety of monitored treatment interruptions in children and highlight the potential of bNAbs to maintain long-term remission.

Regarding the management of the intact proviral reservoir, IMPAACT 2008 assessed the antiviral activity of subcutaneous VRC01 in addition to standard ART in ILHIV, compared to ART alone (55). While no significant differences were observed in the reduction of the intact proviral reservoir, a dose-dependent antiviral effect was identified between the groups, with higher plasma VRC01 concentrations correlating with lower HIV-1 DNA levels. Early reservoir restriction is being further explored in the IMPAACT P1115 trial, a phase I/II study evaluating whether very early ART combined with VRC07-523LS can lead to ART-free remission. By targeting the reservoir before it fully stabilizes, these studies represent a cornerstone in the search for a pediatric functional cure.

These studies have consistently demonstrated that bNAb administration is safe and well-tolerated across in pediatric cohorts. Safety data from different trials have shown that the most common adverse events (AEs) were mild-to-moderate local injection site reactions that included induration, erythema, and tenderness. These Grade 1 and 2 reactions have been reported in up to 90% of infants receiving subcutaneous bNAbs, although typically resolving within 24 h of injection. Conversely, systemic AEs, such as irritability, appetite changes, and sleep disturbances, were rare (6%–20%) and generally mild. While the definitive benefit-risk profile of bNAbs remains to be fully established through ongoing efficacy trials, current clinical evidence positions these biologics as promising therapeutic candidates for HIV-1 prevention, treatment, and potential cure strategies.

## 5 Future trials in children

Building on the proof-of-concept provided by these trials, several initiatives are currently under development to evaluate the neutralization breadth and durability through next-generation molecules, triple-bNAb combinations, and the integration of bNAbs with other immunotherapies. For instance, the PedMab1-ex study will extend the findings of the original PedMab trial by evaluating the safety of ePGT121v1-LS either alone or in combination with VRC07-523LS, providing critical data on the feasibility of different parenteral routes in high-risk infants.

The therapeutic potential of bNAbs will be assessed in the ENABLE trial, funded by Global Health EDCTP3 and European Union under Grant Agreement 101190620, a phase I/II study planned for 2027. This multicenter study will evaluate the safety and preliminary efficacy of subcutaneous ePGT121v1-LS, administered either alone or in combination with VRC07-523LS in ILHIV across multiple African sites. While focusing on establishing virological suppression rates and pharmacokinetic profiles, the trial also incorporates secondary endpoints specifically designed to explore biomarkers of long-term viral remission and the feasibility of an HIV-1 cure in pediatric populations.

Other studies under development will address ART-free remission during ATI, contributing to HIV-1 cure research. The Tatelo Plus (IMPAACT 2042), a phase I/II open-label trial will investigate viral and immune responses to PGT121.414-LS, VRC07-523LS, and PGDM1400LS in CLHIV. This remission strategy will be further evaluated in a pioneering trial, IMPAACT 2039, designed to evaluate the safety and immunogenicity of an HIV T-cell vaccine in combination with the same triple-bNAb cocktail used in the Tatelo Plus. Similarly, the SNOW trial will evaluate the durability of viral control after administration of a single bNAb during ATI in CLHIV.

Collectively, these ongoing and planned studies reflect a transition toward personalized and highly potent interventions. However, despite these advances, significant gaps need to be addressed for the clinical translation of bNAbs. The optimal dosing and PK/PD profiles across various pediatric weight bands are not yet fully defined, and the efficacy of these combinations in viremic infants remains a critical area for future investigation. Ultimately, the successful implementation of these next-generation tools will depend not only on clinical efficacy but also on addressing the manufacturing and logistical barriers that currently limit their global reach.

## 6 The need for trials in children

Despite the disproportionate burden of HIV in the Global South, the clinical evaluation of bNAbs has primarily occurred in the Global North, with study populations predominantly composed of adult males. Of the numerous clinical trials evaluating bNAbs, only a minority have been conducted in high-burden settings, and only a fraction of those have included pediatric cohorts. This exclusion persists despite children representing more than 15% of all AIDS-related deaths while only accounting for less than 5% of all PLHIV (1).

The historical exclusion of CLHIV from clinical trials has been often justified by the complexity of pediatric research. However, delaying the inclusion of children until adult safety is established results in a form of structural inequity, denying this vulnerable population access to life-saving therapeutics (56). This delay is clinically and ethically unacceptable for a life-threatening disease for which mortality remains significantly elevated among infants (10%) (2) during the first year of life, compared to adult cohorts (3%–4%) (57–61), despite early ART initiation.

Beyond the ethical imperative, there are fundamental biological challenges that need to be addressed in the pediatric population. The PK of bNAbs in children are highly dynamic and cannot be simply extrapolated from adult data. Factors such as rapid growth, evolving body fat distribution, and the pediatric immune system significantly alter the half-life and clearance of these monoclonal antibodies. Without dedicated pediatric trials, researchers cannot determine the optimal timing for bNAb initiation relative to ART, the appropriate duration of therapy, or the most effective antibody combinations for early-life immune development. It is the duty of the scientific community to include vulnerable populations in the research pipeline, rather than relying on the off-label prescription of drugs tested exclusively in adults.

To maximize the global impact of bNAbs and ensure they reach the children who need them most, a strategy of intentional early inclusion is required. This proactive approach is essential to address the substantial gaps that currently limit pediatric care, as waiting for post-licensure adult data ignores the physiological and immunological features of infants. Therefore, pediatric clinical development must occur in parallel with adult trials, ensuring that safety and dosing data are established specifically for children without the usual long delay observed with other drugs. This effort must be complemented by the broadening of clinical indications to encompass both treatment and prevention, a step necessary to stimulate the market demand and procurement volumes that justify large-scale manufacturing. Ultimately, achieving true equity necessitates the transfer of bNAb manufacturing technologies to producers within the Global South, securing a sustainable and low-cost supply chain in the regions where the pediatric HIV pandemic remains the most prevalent.

As the field moves toward multi-specific bNAbs and gene-therapy-based delivery systems, the scientific community must ensure that children are not once again left behind. Achieving equity in the bNAb landscape is not only a scientific necessity to explore the limits of HIV-1 cure research but also a moral imperative to dismantle the systemic barriers that sustain the pediatric pandemic.

## 7 Challenges and opportunities

**Figure 1:**
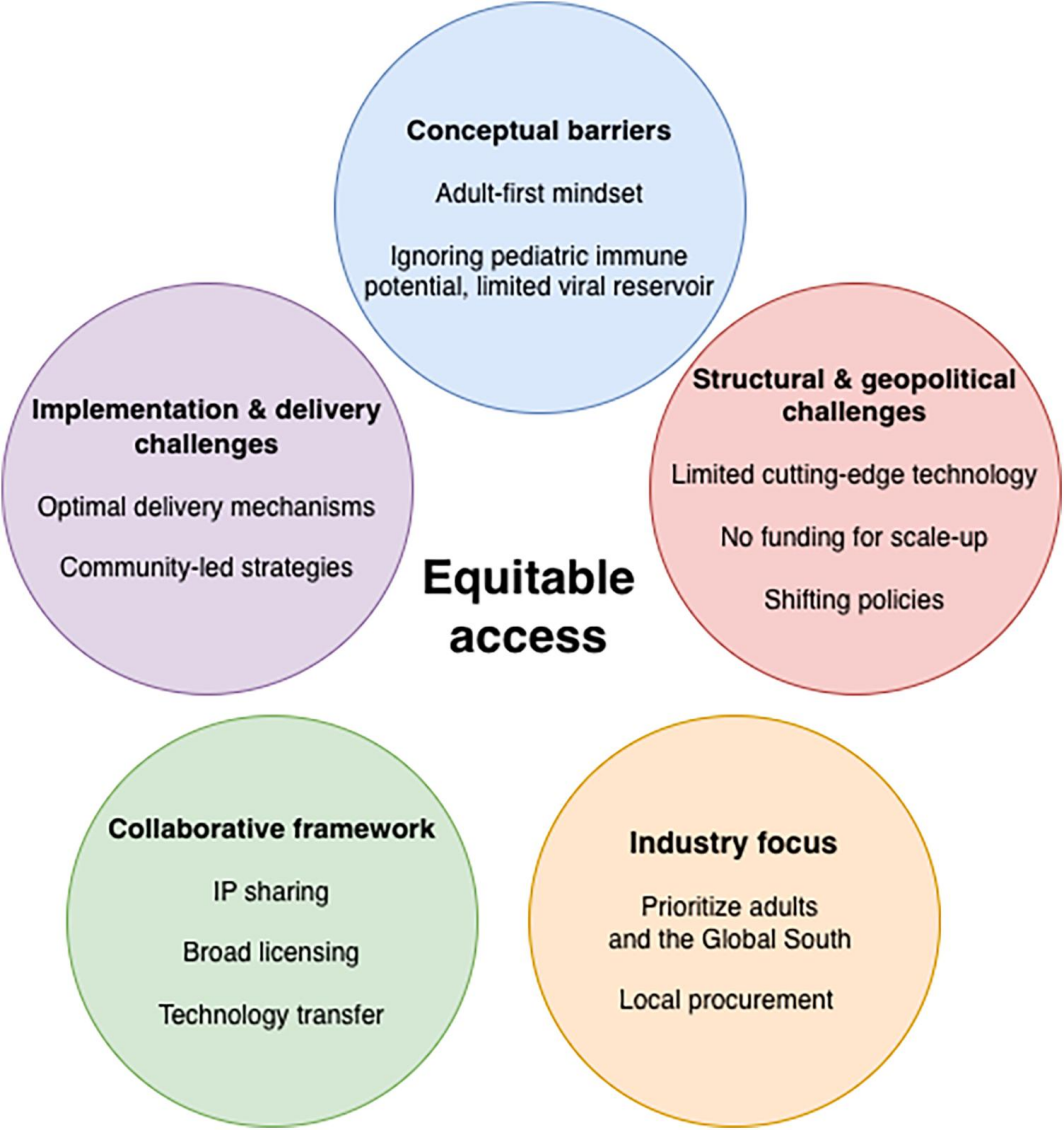
Overall challenges to achieve equitable access of bNAbs in the Global South.

Equitable access is frequently limited by the prevailing view among some researchers that HIV cure strategies should first be achieved in adults before being transferred to children (Figure 1). This perspective fails to account for the unique biological potential of the pediatric immune system. Moreover, children possess an exceptional immune ontogeny and a less diversified viral reservoir that may provide the key to achieving long-term viral remission.

Despite these conceptual barriers, the clinical implementation of bNAbs is constrained by significant structural and geopolitical challenges. The availability of these biologics in the Global South has been historically limited by the lack of cutting-edge technology required for manufacturing and the substantial financial investment required for scale-up. The heavy reliance on a small group of developers leads to a fragile pediatric research pipeline directly impacted by shifting governmental policies and funding reductions. Furthermore, the pharmaceutical industry often prioritizes adult populations or higher-margin markets in the Global North, leaving the 1.4 million children living with HIV in high-burden settings effectively sidelined in the development pipeline.

Transitioning from dependence on foreign agencies to regional procurement requires that countries in the Global South prioritize domestic funding and promote local manufacturing. The recent partnership between IAVI, Minapharm, and ProBioGen, aimed at facilitating the clinical development and manufacturing of bNAbs and vaccines in Africa, demonstrates the feasibility of such regional efforts and will be essential to provide a strategic roadmap and identify operational challenges for future collaborations. Similarly, while production costs are frequently cited as a definitive obstacle to implementation, cost-effectiveness analyses indicate that bNAbs represent a viable intervention in Sub-Saharan Africa, particularly for the prevention of vertical transmission in high-prevalence areas (44). A recent initiative funded by the Gates Foundation and Life Arc identified a group of institutions with potential to develop these monoclonal antibodies at exceptionally low cost, challenging the narrative that these tools are inherently unsuitable for resource-limited settings.

Addressing the remaining obstacles to obtain equitable access requires a collaborative action plan that engages governments in the Global South, stakeholders, funders, and biopharmaceutical manufacturers to facilitate intellectual property sharing, broad licensing, and technology transfer (62). The HIV Peri- and Post-Natal (PNP) task force exemplifies this collaborative approach (63). It has established a robust framework for pediatric bNAb prophylaxis, that has been translated into the development of the ENABLE trial where the treatment potential of bNAbs will be evaluated in ILHIV. By optimizing production and increasing visibility in long-term demand, the global health community can lower prices, mitigate financial risks for local manufacturers, and incentivize large-scale production while supporting the development of products that meet WHO-preferred characteristics.

The scaled use of these products will pose new delivery and implementation challenges that require prompt attention. Establishing optimal delivery mechanisms will require early catalytic introduction programs and implementation research conducted in close partnership with national stakeholders. To ensure that adoption responds to regional priorities, it is essential to work alongside local communities to raise awareness and dismantle the social barriers that define the pediatric pandemic. By aligning regulatory pathways with community-led implementation strategies, the scientific community can ensure that these innovative products are not only available but effectively integrated into the global pediatric HIV response.

## 8 Conclusion

Clinical trials in children have demonstrated the safety and efficacy of bNAbs in this vulnerable population. Therefore, their implementation constitutes an ethical obligation to prevent the further widening of global health disparities. To address this crisis, regulatory agencies, researchers, and the pharmaceutical industry must prioritize pediatric cohorts and integrate them into the earliest stages of the bNAb development pipeline, moving beyond the historical adult-first paradigm. bNAbs represent the next frontier in pediatric HIV care, offering a critical alternative from the burden of daily oral therapy and a unique biological window for long-term viral remission. However, the potential of these biologics will only be reached, when manufacturing equity, cost-effectiveness, regulatory harmonization has been achieved in the Global South. Prioritizing pediatric bNAb development is a fundamental requirement to explore the limits of HIV-1 cure research and a moral imperative to ensure that the most vulnerable children are not left behind in the next generation of medical innovation.
